# Application and progress of advanced eye movement examinations in cognitive impairment

**DOI:** 10.3389/fnagi.2024.1377406

**Published:** 2024-04-17

**Authors:** Qi Leng, Bo Deng, Yi Ju

**Affiliations:** ^1^Department of Neurology, Beijing Tiantan Hospital, Capital Medical University, Beijing, China; ^2^Clinical Center for Vertigo and Balance Disturbance, China National Clinical Research Center for Neurological Diseases, Beijing, China

**Keywords:** advanced eye movement, examination, cognitive impairment, saccades, dementia

## Abstract

The worldwide incidence of cognitive impairment is escalating, yet no effective solutions for these afflictions have been discovered. Consequently, the importance of early identification and immediate intervention is heightened. Advanced eye movements—a form of voluntary eye movements that includes anti-saccades, memory-guided saccades, predictive saccades, pro-saccades and gap/overlap saccades, mediated by the cerebral cortex and subcortical pathways reflect cognitive levels and functions across different domains. In view of their objectivity, reproducibility, and non-invasive characteristics, advanced eye movement examination possesses significant prospective utility across a wide range of cognitive impairment. This paper extensively reviews various models associated with advanced eye movement examinations and their current applications in cognitive impairment such as Alzheimer’s disease, Lewy body dementia and frontotemporal dementia. Advanced eye movement examination can serve as a biomarker for early screening diagnosis and research on cognitive impairment. In the future, combining advanced eye movement examination with neuropsychological scale assessment and other diagnostic methods may contribute to further early identification of these types of diseases.

## 1 Introduction

The prevalence of diseases characterized by cognitive impairment (cognitive impaired diseases) or diseases accompanied by cognitive impairment will increase globally with the aging of population, and according to the World Health Organization (WHO), it is predicted that there will be about 78 million people with dementia in 2030, and will reach 139 million in 2050 (World Health Organization, 2021). Cognitive impairment refers to a subjective or objective decline in cognitive functions such as memory, comprehension, reasoning, attention. Currently, clinical diagnosis of cognitive impairment mainly relies on neuropsychological scale, and examinations such as positron emission tomography (PET). Biological markers such as cerebrospinal fluid Aβ and Tau protein can also assist in clinical diagnosis (Albert et al., 2011). However, tests above have limited the widespread promotion in the population due to the time-consuming, expensive and invasive conditions, respectively.

Eye movements refer to the voluntary or involuntary eye rotations emitted by humans to ensure that the retina can image the outside world in a stable manner. Eye movements are not only the way a person interacts with the external world, but can also reflect internal cognitive processes. Various paradigms have been designed to elicit, monitor, and analyze eye movement by giving specific instructions to subjects, and are used as a novel objective, reproducible, and non-invasive examination method for scientific research and clinical applications in cognitive impairment and psychiatric disorders. Eye movement examination can be widely used as a valid complement to neuropsychiatric-psychological scale assessment (Lin et al., 2023).

**Figure 1:**
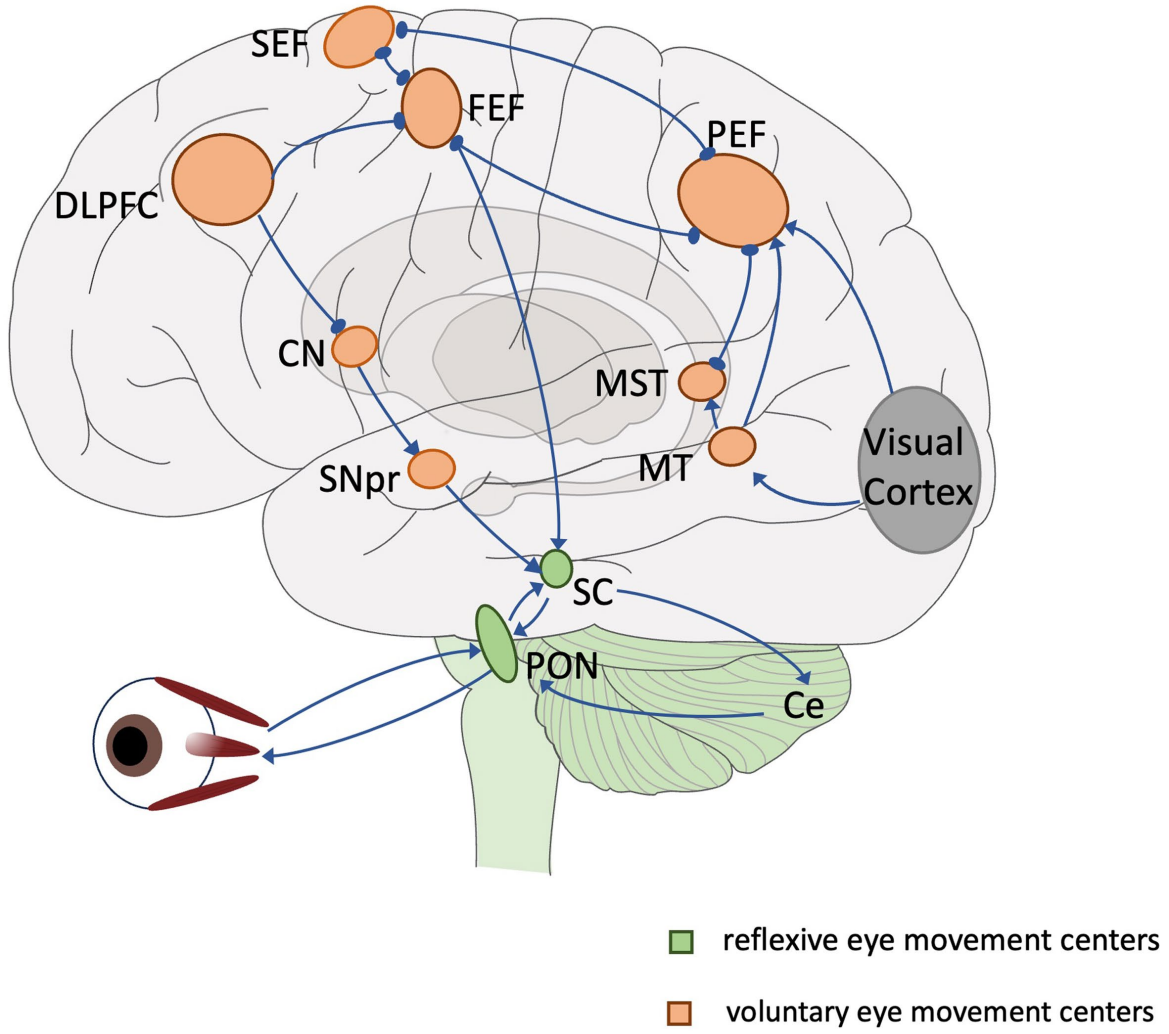
Overview of the eye movement neural network. Lines and arrows indicate relationships of individual eye movement centers. Green: reflexive eye movement centers, orange: voluntary eye movement centers. SEF, supplementary eye field; FEF, frontal eye field; PEF, parietal eye field; DLPFC, dorsolateral prefrontal cortex; CN, caudate nucleus; MT, middle temporal region; MST, medial superior temporal region; SNpr, substantia nigra pars reticulata; SC, superior colliculus; PON, pontine nucleus; Ce, cerebellum.

Eye movements are often categorized according to functional differences in different contexts: vestibulo-ocular reflex, optokinetic response, fixation, convergence, saccade and pursuit (Kassavetis et al., 2022). Eye movements can also be categorized according to their anatomical pathways into low-level reflexive eye movements and high-level voluntary eye movements. The centers of low-level reflexive eye movements are mainly located in the midbrain. Voluntary eye movements are controlled by cortical–subcortical eye movement centers (referred to as advanced eye movements) with the involvement of cortical and subcortical pathways, which involve more cognitive processing and are able to reflect the level of cognition and the functioning of different cognitive domains (Figure 1). These voluntary eye movements include antisaccade (Hallett, 1978) memory-guided saccade (Bruce and Goldberg, 1985), predictive saccade (Stark et al., 1962), pro-saccade (Deubel, 1996), gap/overlap saccades (Saslow, 1967). Among multiple types of eye movements, saccade is easier to monitor and quantify, and also quite editable (only the temporal and spatial order of visual signals needs to be changed or the subject needs to be instructed). Therefore, they are frequently applied in cognitive function assessments.

Saccade is a rapid jumping eye movement that causes a visual target to be rapidly imaged in the central retinal sulcus, allowing immediate visualization of the target. The superior colliculus of the midbrain is the key structure controlling the emission of all saccades, which is responsible for the determination of the location of the saccade target and ensures the accuracy of the saccadic direction and the termination position. The information received prior to efferent excitation in the superior colliculus can be broadly categorized into two situations: (1) mainly consists of exogenous information, i.e., the location, size, shape, and luminance of the visual signal, which is primarily involved in attentional processes. The neural circuits involved in the formation and emanation of saccade are mainly concentrated at the brainstem level. (2) Information is largely endogenous, which is in need of more cognitive processes, i.e., higher cognitive workloads (e.g., in antisaccades, inhibitory control of the visual signal, initiation of saccade stimuli at the mirror position of the saccade). These processes require the involvement of cortico-subcortical pathways, such as the dorsolateral prefrontal cortex (DLPFC). Then, we call the saccades toward an exogenous unanticipated signal (including visual, auditory, tactile, etc.) that are induced by that signal reflexive saccades; and the subjective purposive or selective saccades are called voluntary saccades, which belong to the advanced eye movements mentioned before. In practical application for testing cognitive functions, researchers often add different types or intensities of cognitive workloads to reflexive saccades, and obtain a variety of examination paradigms.

Parameters used in saccade include: (1) Latency or saccade reaction time (SRT): Examining the process of a single saccade movement, the information from endogenous and exogenous sources needs to be integrated between the appearance of the saccade target and the issuance of the saccade movement, which is called the saccade latency (or SRT). It includes the processing of receptor information (mostly visual information), drawing attention, selection of the saccade target, decision to execute, encoding of the movement signal, and the efferent process. Different advanced eye movement examination paradigms differ mainly by affecting the endogenous information processing process and, consequently, the SRT. (2) Saccade acceleration: The rate of change of the eye rotation speed over time was defined as the acceleration. (3) Saccade peak velocity: During the process of switching gaze points, the speed of eye movement gradually increases. And the moment when the rotation velocity reached 30°/s was regarded as the beginning of the saccade, and the maximum instantaneous velocity that could be reached was defined as the peak velocity (Allman et al., 2010). (4) Saccade amplitude: After reaching the peak velocity, the eye rotation speed gradually slowed down until it stabilized at the saccade target, and we regarded the moment when the instantaneous eye rotation velocity decreased to 5% of the peak velocity as the end of the saccade (Goldberg et al., 2002). The maximum angle of eye rotation from the beginning to the end of the saccade is defined as the saccade amplitude. (5) Saccade gain: The ratio of the instantaneous velocity of eye rotation to the rate of movement of the saccade target throughout the saccade is defined as the saccade gain.

In this review, we will present in turn the characteristics, intrinsic anatomical pathways, as well as the current and prospective applications of above advanced eye examinations in cognitive impaired disease (Table 1, some other eye movement parameters have also been supplemented in it).

**Table 1:** Summary of clinical features of advanced eye movement in cognitive disorders.

	Measure	AD	DLB	FTD	PD	PDD	PSP	MSA	ALS	MS
Pro-saccades	Latency	↑↑	–	↑	–	↑↑	↑	↑↑	↓	↑
	Gain	↓↓		–	↓	↓↓	↓	↓		↓
Antisaccades	Latency	↑↑	↑	↑	↑	↑↑	↑	–	↑	↑
	Errors	↑	↑	↑	↑	↑↑	↑	↑	↑	↑
Memory-guided saccades	Latency		↑	↑	↑		–		↑	↑
	Errors		↑	↑	↑		↑		−/↑	↑
Predictive saccades	Rate	↓↓	↓	↓	↓	↓↓				↓
	Latency	↑↑	↑	↑		↑				
	Gain	↓				↓				

This table summarizes the advanced eye movement manifestations of cognitive impairment observed in studies. AD, Alzheimer’s disease; PD, Parkinson’s disease; PDD, Parkinson’s disease dementia; DLB, dementia with Lewy bodies; FTD, frontotemporal dementia; PSP, progressive supranuclear palsy; MSA, multiple system atrophy; CBD, corticobasal degeneration; ALS, amyotrophic lateral sclerosis; MS, multiple sclerosis; −, no difference from control subjects; ↑, increased; ↑↑, greatly increased; ↓, decreased; ↓↓, greatly decreased.

This review of articles extracted from PubMed with several search terms, such as “eye movement,” “eye tracking,” “saccades,” “nystagmus,” “eye gaze,” “ocular fixation,” “oculomotor,” “antisaccades,” “memory saccades” “predictive saccades,” “anticipatory saccades,” “pro-saccades,” “gap saccades,” “overlap saccades,” “cognitive impairment,” “Mild cognitive impairment,” “MCI,” “Alzheimer’s disease,” “AD,” “Lewy body dementia,” “DLB,” “Frontotemporal dementia,” “FTD,” “Parkinson’s disease,” “PD,” “Progressive supranuclear palsy,” “PSP,” “Multiple system atrophy,” “MSA,” “Amyotrophic lateral sclerosis,” “ALS,” “Multiple sclerosis,” and “MS,” the articles were selected based on their educational value.

## 2 Definition and anatomical pathways of advanced eye movements

**Figure 2:**
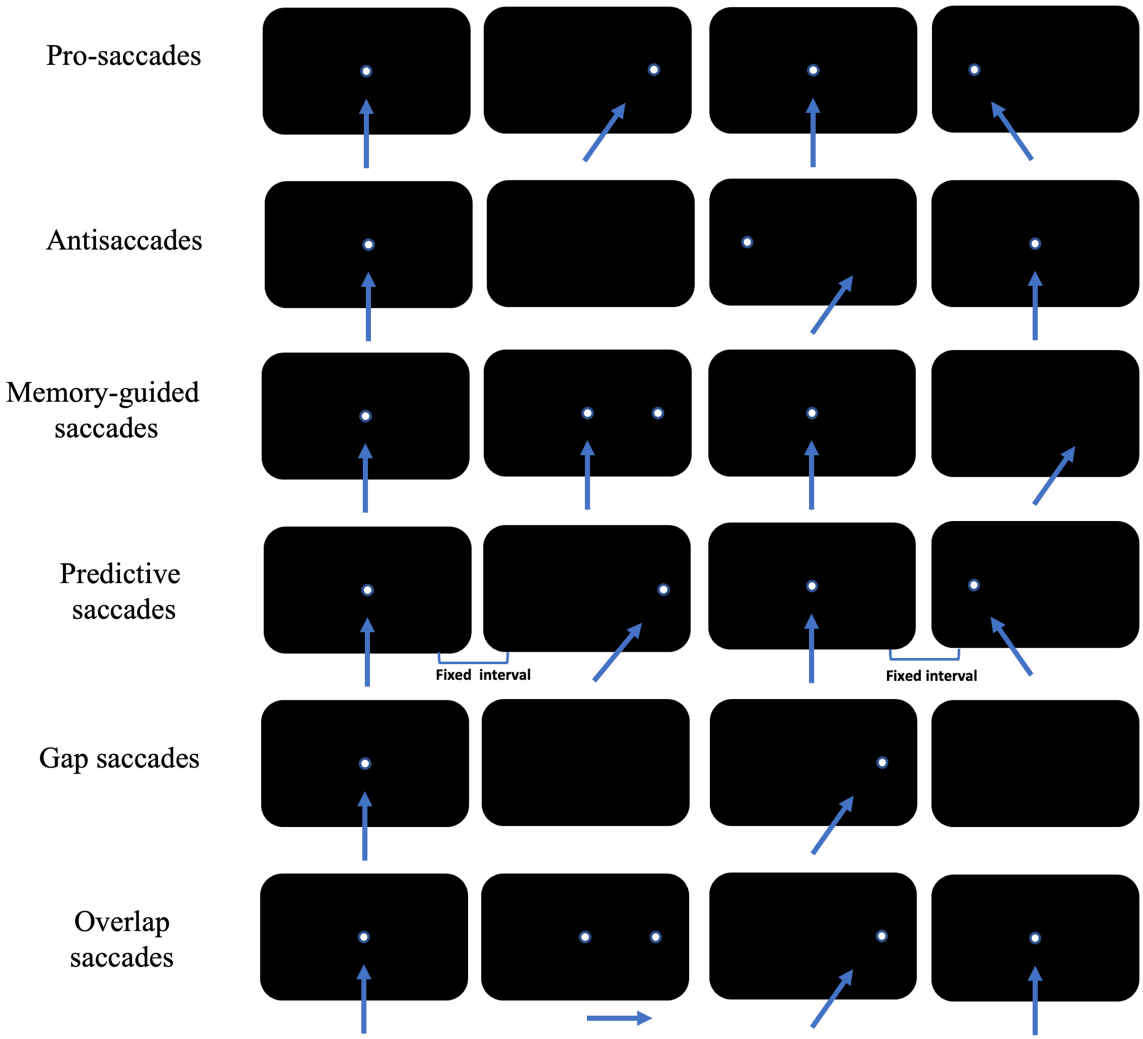
Schematic diagram of the eye movement examination method. The arrow shows the direction of subjects’ eye movement, and the white dot is the position of visual targets. Each row of black rectangles from left to right shows sequential displacement of visual target across time.

Saccade is a rapid jumping eye movement that causes a visual target to be rapidly imaged in the central retinal sulcus, allowing immediate visualization of the target. Depending on the examination paradigm, cognitively relevant saccades include antisaccade, memory-guided saccade, predictive saccade, pro-saccade, and gap/overlap saccade (Figure 2).

### 2.1 Pro-saccades

Pro-saccades require subjects to first gaze at fixation stimulus located in the center of the visual field, and at the same time as the fixation stimulus disappears, saccade stimulus appears elsewhere in the visual field. Subject is required to shift the gaze point to the newly appearing saccade stimulus as quickly and accurately as possible.

The pro-saccade paradigm is the advanced eye movement examination paradigm with the least cognitive workload, and requires subjects to initiate saccades as quickly and accurately as possible. The saccade emitted in this condition is dominated by exogenous visual information, with varying extents of involvement of endogenous information (e.g., quick judgment of the necessity of this saccade), with a saccade reaction time of about 200 ms. Considering there is a significant effect of cognitive functioning on their latencies, so it is necessary to examine the performance of the pro-saccade paradigm in patients with cognitive impairments. Pro-saccade involves the cooperative work of several regions in the higher cortex of the brain, such as prefrontal cortex (PFC), FEF, SEF, PEF, etc. (Calancie et al., 2022).

### 2.2 Antisaccades

In antisaccade tasks, subjects first gaze at the fixation stimulus located at the median of the visual field. At the same time as the fixation stimulus disappears, the saccade stimulus will randomly appear somewhere in the visual field, and subjects will be asked to look as quickly as possible to the centrosymmetric position of the saccade stimulus about the median of the visual field.

In antisaccades, subjects first need to inhibit reflexive saccades toward the saccade stimulus (failure of which is judged to be an error saccade) (Slovin et al., 1999), and then convert the acquired saccade position information into voluntary saccade signals toward the opposite direction. In this process there are two competing response processes—unconditioned response induced by visual signals and active response induced by voluntary movement signals, i.e., antagonism between exogenous and endogenous information (Munoz and Everling, 2004). Thus, the antisaccade paradigm may reflect inhibitory control and voluntary movement functions. In practice, the ratio of the number of erroneous saccades to the number of all saccades is defined as the antisaccade error rate, and the saccade emitted successively in the correct direction after an erroneous saccade is referred to as a corrective saccade. These parameters are commonly used to evaluate the subject’s antisaccade performance.

Antisaccades are more difficult to understand and perform because of the additional process of “inhibition” as compared to pro-saccades. In healthy adult subjects, the latency of error saccades was comparable to that of pro-saccades, with an error rate of about 20–25%, while the latency of corrective and antisaccade saccades was prolonged by about 100 ms (Munoz and Everling, 2004).

Functional areas of the brain involved in anti-saccade include the frontal eye field (FEF), supplementary eye field (SEF), dorsolateral prefrontal cortex (DLPFC), parietal eye field (PEF), and anterior cingulate cortex (ACC) (McDowell et al., 2008; Cieslik et al., 2016). It has been shown that damage to the DLPFC and cingulate eye fields (CEF) leads to increased anti-saccade errors, and damage to the FEF leads to increased anti-saccade latency (Cambron et al., 2011).

### 2.3 Memory-guided saccades

In memory-guided saccade tasks, subjects first look at the fixation stimulus located in the median of the visual field, and the saccade stimulus will randomly appear somewhere in the visual field before the fixation stimulus disappeared. Then they will be asked to maintain their gaze at the median stimulus and to memorize the location of the saccade stimulus. The fixation stimulus will disappear after a certain period of time (often 200 ms) after the disappearance of the saccade stimulus. At this point, subjects will be to look as quickly and accurately as possible to the location where the saccade stimulus has appeared. Successful completion of the memory-guided saccade paradigm also requires subjects to inhibit reflexive saccades while briefly memorizing the spatial location of the saccade stimulus. In practice, the concept of accuracy is often introduced to further characterize a subject’s memory-guided saccade performance, i.e., the extent to which the gaze point is in agreement with the position of the saccade target at the end of a saccade, often measured as the ratio of the magnitude of the saccade to the angular distance between the two gaze points.

Memory-guided saccades are associated with a higher cognitive workload than pro-saccades and antisaccade saccades, with a significant decrease in saccade accuracy, prolonged latency and reduced peak velocity in healthy adult subjects (Hutton, 2008). Memory-guided saccade is one of the effective ways to detect spatial working memory. Studies have shown that FEF, SEF, and PEF play important roles in spatial working memory generation (Xiao et al., 2006; Chen et al., 2016), and extraretinal signals, such as vestibular signals, can also assist in the generation of memory-guided saccades (Li et al., 2005). It can reflect the brain’s ability to store information about the spatial location of objects, which in turn assesses working memory, and attention (Suzuki and Gottlieb, 2013).

### 2.4 Predictive saccades

Predictive saccades require subjects to first gaze at a fixation stimulus that is located at the center of the visual field, and at the same time that the fixation stimulus disappears, the saccade stimulus will appear in the visual field at two fixed positions at fixed intervals in turn. Subjects are required to shift their gaze to the newly appearing saccade stimulus as rapidly and accurately as possible. This endogenous saccade, produced without the assistance of direct visual information, is called predictive saccade (Calancie et al., 2022).

The difference between the predictive saccade paradigm and the pro-saccade paradigm is that the frequency and location of the saccade stimulus are fixed and completely predictable (meaning that they can be predicted in both time and space). In addition to receiving information about changes in the position of the stimulus, subjects can gradually integrate information about the frequency and positional patterns of stimulus changes and output endogenous commands to act on saccades. The results showed that after 4–6 stimulus transformations, the latency of the saccades began to shorten significantly, preceding the appearance of the saccade stimulus (Okada et al., 2022). Generally, saccades with a latency less than 80 ms are defined as predictive saccades (Tokushige et al., 2021). On this basis, the predictive saccade percentage, i.e., the ratio of the number of predictive saccades to the number of all saccades, is used to evaluate the subject’s predictive saccade performance.

Studies have shown that the FEF, SEF, DLPFC (Lee et al., 2016; Calancie et al., 2022), and cerebellum (Tanaka et al., 2021) play important roles in the predictive saccade process. The significance of predictive saccade examination is that the movement of the visual target is predictable, and subjects can predict the location of the target based on prior experience, thus reflecting their cognitive functions related to establishing and maintaining internal symbols in response to environmental events, such as motor planning and spatial working memory in executive function.

### 2.5 Gap/overlap saccades

If the time difference between the disappearance of the fixation stimulus and the appearance of the saccade stimulus is changed, a new perspective can be added to the original paradigm. In practice, two additional conditions, “gap” and “overlap,” are often imposed on the basis of the pro-saccade paradigm.

The gap condition, in which a fixed time is spaced between the disappearance of each fixation stimulus and the appearance of the saccade stimulus, requiring subjects to look as quickly and accurately as possible at the saccade stimulus. The gap in this condition provided some temporal predictability for the next saccade initiation, while allowing subjects to disengage from the gaze condition earlier and be able to be oriented more rapidly to new visual signals (Delerue et al., 2010). The gap condition induced a significantly shorter latency for saccades and a significantly higher proportion of express saccades than pro-saccades, a phenomenon known as the gap effect.

In the overlap condition, the fixation stimulus will coexist with the saccade stimulus for a period of time before disappearing. Subjects will continue to gaze for a longer period of time, visual attention is continuously occupied and difficult to be oriented to the new stimulus, and the latency of the saccade stimulus is significantly prolonged compared to pro-saccade, a phenomenon known as the overlap effect.

Gap and overlap saccades mainly detect the function of FEF and areas such as PEF and superior colliculus (SC) associated with visual attention shifts (Mosimann et al., 2005), and can be used for the assessment of attention level.

## 3 Clinical implications of different eye movement paradigms

Currently, the evaluation of cognitive level mainly relies on neuropsychological scales, such as Minimum Mental State Examination (MMSE) and Montreal Cognitive Assessment (MoCA), which can assess the overall cognitive level (the higher the score, the closer the cognitive level is to the normal level), but these scales sometimes have the problem of insufficient differentiation. In this section, we will sequentially introduce the application of advanced eye movement examination in mild cognitive impairment, the three most common dementia-causing diseases, and other neurodegenerative diseases accompanied by varying degrees of cognitive impairment, with a view to providing new ideas for evaluating cognitive level and diagnosing cognitive impaired diseases.

### 3.1 Mild cognitive impairment

Mild cognitive impairment is broadly defined as the subjective and objective presence of manifestations of cognitive impairment, with the ability to still live alone, but a level of cognitive impairment that is not yet diagnostic of any of the types of dementia whose etiology includes, but is not limited to Alzheimer’s disease, Parkinson’s disease and dementia with Lewy bodies (Albert et al., 2011). On this basis, it is further classified, depending on the cognitive domains that are impaired, into amnestic mild cognitive impairment (aMCI), with significantly impaired memory (which is thought to be a pre-existing stage of AD), and nonamnestic mild cognitive impairment (naMCI) with significant impairment in other cognitive domains such as attention and executive function.

It is currently believed that there are multiple transition states between the aging population with normal cognitive levels and the demented group, i.e., subjective cognitive decline and mild cognitive impairment, and advanced eye movement examination studies of the former are lacking (Petersen et al., 2014). But it is increasingly recognized that advanced eye movement examination as a non-invasive test helps in the early identification, staging and differentiation of patients with MCI from other diseases by quantifying the level of cognition (Wilcockson et al., 2019).

Prolonged latency, decreased accuracy, decreased error correction, and decreased gain in anti-saccades in patients with MCI, and anti-saccade error correction correlates with MMSE scores, which may be due to decreased frontal lobe function that results in impaired inhibition, working memory, and other functions of patients. Abnormalities in these functions often precede clinical measures and structural imaging abnormalities. Reaction times and error rates of anti-saccade work better in identifying subjects with normal cognitive level or with MCI than pro-saccade, which is more close to reflexive saccade process, whereas anti-saccades require inhibition of reflexive saccade and initiation of voluntary saccade, a process that often involves more cognitive processes. The anti-saccade can also be used to differentiate aMCI and naMCI (Koçoğlu et al., 2021). The results of some other studies showed that from healthy controls to naMCI, aMCI, and AD, their anti-saccade accuracy, anti-saccade error correction rate and pro-saccade latency gradually declined, anti-saccade latency gradually lengthened, and this abnormality may be closely related to the difference in the functional areas involved in patients with aMCI and naMCI and the involvement or not of the hippocampus and the internal olfactory cortex (Wilcockson et al., 2019). In addition, the latency length and error rate of pro-saccades and gap/overlap saccades can be used as one of the indicators to differentiate between healthy, MCI and AD groups, with MCI patients having a prolonged latency compared to healthy controls and AD patients having a significantly longer latency than MCI patients (Chehrehnegar et al., 2022; Opwonya et al., 2022).

### 3.2 Alzheimer’s disease

Alzheimer’s disease is the most common cause of dementia and is characterized by an insidious progression of total cognitive decline, including memory, judgment, and attention (especially memory) (Scheltens et al., 2021). Patients with Alzheimer’s disease are likely to suffer executive function and attention deficits much earlier than average (Rozzini et al., 2007), leading to abnormal advanced eye movement examination results, which are closely related to the severity of the disease and the degree of cortical atrophy. For example, AD patients have decreased accuracy and prolonged latency of memory-guided saccades (Lage et al., 2020), longer latency of anti-saccades than healthy controls, significantly higher error rate of anti-saccades, and lower rate of error correction (Kahana Levy et al., 2018). Patients with AD have a decreased percentage of predictive saccades and a longer latency compared to healthy controls (Opwonya et al., 2022). The results of the memory-guided saccades, anti-saccades, and predictive saccades examinations indicate that AD patients are unable to inhibit reflexive saccades, a phenomenon that may occur as a result of damage to the DLPFC and ACC and insufficient top-down inhibition of neurons in the FEF and SC prior to target emergence. Gap saccade latencies are shorter in both patients and controls than young controls, and this difference is more significant between age groups, but not between old controls and AD patients of the same age (Crawford et al., 2013).

In addition, recent meta-analysis results have shown prolonged pro-saccade latency and decreased saccade speed in AD patients compared to healthy controls, and no significant change in saccadic gain was found compared to controls. The pro-saccades latency was negatively correlated with MMSE scores, and it has been suggested that the increased latency is associated with a decrease in bilateral parietal, occipital, and right temporal lobe volumes (Kahana Levy et al., 2018).

### 3.3 Dementia with Lewy bodies

Dementia with Lewy bodies is the second most common cause of dementia, characterized by fluctuating levels of cognition (especially attention), mild motor deficits, and vivid hallucinations. Large numbers of Lewy bodies are often found on histologic examination of the cerebral cortex and brainstem in such patients (McKeith et al., 2017). There was no significant difference between DLB and AD patients in anti-saccades error rate and latency, but the error correction rate was significantly lower in the DLB group than AD group. Patients with DLB had prolonged predictive saccade latency, decreased gain, and decreased percentage of predictive saccades compared with AD patients (Kapoula et al., 2010). In both pro-saccade and gap/overlap saccades, DLB patients had longer saccade latency and reduced saccade gain than AD patients. This difference may be due to the coexistence of cortical and subcortical neurodegeneration in DLB patients, resulting in inferior performance on advanced eye movement examination compared to AD patients, who are predominately influenced by cortical neurodegeneration (Mosimann et al., 2005).

### 3.4 Frontotemporal dementia

Frontotemporal dementia is the third most common cause of dementia and refers broadly to a large group of disorders characterized by progressive, insidiously progressing behavioral abnormalities, reduced executive functioning, or reduced language functioning (Bang et al., 2015). Behavioral variant of frontotemporal dementia (bvFTD) associated with frontal lobe atrophy is the most common subtype of frontotemporal dementia in clinical practice. Compared with healthy controls, patients with bvFTD have significantly reduced anti-saccades accuracy, correction rate, and memory-guided saccade accuracy. Although such patients had a similar rate of anti-saccades error as AD patients, their anti-saccades correction rate and memory-guided saccade accuracy were better than those of the AD group. bvFTD group had a significantly longer latency period for memory-guided saccade and anti-saccades examination, but their performance was still better than that of AD patients. Executive dysfunction due to impaired frontal lobe function may be the main cause of these abnormalities. Another study found a reduced percentage of predictive saccades and a prolonged latency period in bvFTD patients, which researchers suggested was related to their emotional apathy, increased blinking, distraction, and BG lesions (Mosimann et al., 2005; Deravet et al., 2021).

### 3.5 Parkinson’s disease

Parkinson’s disease is a common and complex neurological disorder characterized by the early and massive death of dopaminergic neurons in substantia nigra pars compacta (SNpc). The clinical manifestations are dominated by symptoms of motor deficits such as tremor and postural and gait abnormalities, and cognitive impairments in this group of patients can appear in early stage of the disease (within 5 years) (Kalia and Lang, 2015). Patients with early PD exhibit prolonged anti-saccades latency and decreased accuracy (Li et al., 2023). Increased anti-saccades latency in patients with PD is associated with postural gait abnormalities and may be a marker for the development of a frozen gait within 5 years (Gallea et al., 2021). Memory-guided saccade latency is prolonged and amplitude decreased, which is decreased more significantly than prosaccade. These evidences suggest the difficulty of intentional saccades initiation in PD patients. Moreover, the percentage of predictive saccades is reduced in PD patients compared to normal subjects, and their performance is more similar to “following” the target than to making “predictions,” suggesting that PD patients have an increased reliance on visual information input during predictive saccades (Bronstein and Kennard, 1985). Interestingly, intentional saccades were mostly affected in the early stages of PD while reflexive saccades were preserved, which may be due to the fact that reflexive saccade signals can project directly from the parietal cortex to SC saccade-related neurons, bypassing the basal ganglion (BG) region (Gorges et al., 2014; Stuart et al., 2019).

About 25–30% of PD patients develop Parkinson’s disease dementia (PDD) (Aarsland et al., 2017). As the patients’ cognitive level declines, the eye movement examination may show a gradual decrease in saccade amplitude and prolonged latency. Compared with AD patients, PDD patients have worse performance in some eye movement paradigms, such as: prolonged latency and increased error rate of anti-saccades, prolonged latency and decreased percentage of predictive saccades, and prolonged latency and decreased gain of pro-saccades. These abnormalities may be related to the involvement of both its cortical and subcortical oculomotor circuits (Mosimann et al., 2005).

### 3.6 Progressive supranuclear palsy

PSP is a clinical syndrome characterized by supranuclear oculomotor paralysis, postural instability, and mild dementia, and its actual clinical manifestations are far more complex than described in the definition (Williams and Lees, 2009).

Currently, video-oculographic (VOG) are often used to diagnose PSP in patients with slowed vertical and horizontal sweeps and asymmetry in vertical saccade velocities (Herwig et al., 2021; Quattrone et al., 2022). In particular, it has been shown that square-wave jerks (SWJ) is present in about 60% of PSP patients. SWJ is a prominent feature of PSP, and SWJ as an involuntary eye movement is a pair of small (<5 deg) saccades, which can potentially serve as an indicator for diagnosis (Zachou et al., 2024).

In advanced eye movement, Patients with PSP have prolonged latency, increased error rates, and decreased self-correction rates for anti-saccades (Garbutt et al., 2008). In memory-guided saccades, the latency of PSP patients was not significantly different from that of healthy controls, but the error rate of memory-guided saccades was significantly increased, and this difference became more and more significant with the progression of the disease (Terao et al., 2013). There may be extensive pathological changes in the brain in PSP patients, including the BG responsible for oculomotor initiation and inhibition and the DLPFC (Wright et al., 2022), which still need to be further explored.

### 3.7 Multiple system atrophy

Multiple system atrophy is a neurodegenerative disease characterized by neuronal degeneration and gliosis in multiple parts of the central nervous system (substantia-nigra-striata, olivary-pontine-cerebellum, etc.), with varying levels of cognitive impairment depending on the site of involvement (Poewe et al., 2022). Features of eye movements in patients with MSA include excessive SWJ, hypometria of saccades, impaired vestibulo-ocular reflex (VOR), and the presence of nystagmus (Kassavetis et al., 2022; Zachou et al., 2024). As for the advanced eye movements, increased anti-saccades error rate with prolonged pro-saccades latency can be used as a marker for early differentiation between PD and MSA, with both PD and MSA patients having higher anti-saccade error rates than healthy controls (Brooks et al., 2017). Only a few studies have applied advanced eye movement examination to MSA, and more patients could be included in further studies in the future.

### 3.8 Amyotrophic lateral sclerosis

ALS is a degenerative motor neuron disease that can involve all segments of the spinal cord, but clinical studies have found that 35–50% of patients with early ALS may be accompanied by varying degrees of cognitive and behavioral deficits and other manifestations of frontotemporal lobe involvement (Feldman et al., 2022). Due to executive dysfunction, ALS patients are often unable to inhibit reflexive saccades, resulting in elevated error rates and prolonged latencies in anti-saccades (Tao et al., 2020), and studies have also confirmed that increased anti-saccades error rates in ALS correlate with reduced DLPFC activity (Poletti et al., 2021). Memory-guided saccades in ALS patients have prolonged latencies and increased error rates compared to normal subjects. Impairment of PFC, FEF, and PEF all results in prolonged latency of the memory-guided saccade. Neuropsychological assessment results also confirmed the presence of frontal lobe damage in ALS patients, but the possibility of concomitant parietal lobe involvement in patients cannot be excluded (Zaino et al., 2022), future studies still in need to confirm this.

### 3.9 Multiple sclerosis

Multiple sclerosis is an autoimmune demyelinating disease that involves the brain and spinal cord, causing a blockage of excitatory transmission, resulting in a range of symptoms such as lethargy, sensory abnormalities, memory loss, and slowed thinking (Marcus, 2022). An increasing number of studies have suggested that cognitive dysfunctions such as attention, executive function, and memory may be present in patients with MS. In advanced eye movement examinations, MS patients often exhibit prolonged latency of anti-saccades and memory-guided saccades, increased error rates, and decreased spatial accuracy (Fielding et al., 2009a,b). However, these results have been hardly reproduced, which may be due to the heterogeneity of the distribution of white matter lesions in MS patients, and the eye movement abnormalities may be due to the involvement of the structure of the oculomotor loop in white matter lesion. The characteristics of the disease itself make it difficult to reproduce the experimental results in different laboratories, and are also related to the small sample size of most studies (García Cena et al., 2022).

## 4 Discussion

In this paper, we review studies of advanced eye movement examination applied to cognitive impairment groups. The concept of advanced eye movements distinguish it from reflexive eye movements, with the aim of highlighting some specific types of eye movements from the complexity and variety of eye movement types.

These movements involve multiple cortical–subcortical regions from planning, encoding to emitting. The essence is the synergy of the functions of the cognitive domains corresponding to these brain regions; with normal levels of functioning of these cognitive domains, eye movements can be executed successfully. A large number of previous studies have examined the functional levels of various cognitive domains in healthy and cognitive impaired populations by designing eye movement examination paradigms with different types of cognitive workload attached to them. The eye movements in these different paradigms, from planning and encoding to the emission of eye movement information, involve multiple cortical–subcortical areas. Essentially, this is a coordinated action of the cognitive domain functions corresponding to these brain regions; under the premise of normal functioning in these cognitive domains, eye movements can be smoothly executed.

This review summarizes the most frequently used paradigms, introduce the basic forms of these paradigms, the basic parameters, the performance of the tests in healthy populations, and the anatomical pathways involved behind them. These paradigms are far from being the full extent of advanced eye movements, and researches have been done to improve upon them, such as adjusting their predictability by varying the number of fixation positions of saccade stimulus or the regularity of the time of their appearance, or adding the task of memorizing the color or shape of a saccade stimulus to the memory-guided saccade. But these variations have not yet been extensively studied, and the cognitive workload is higher than in the original paradigm, which reduces to a certain extent its screening effectiveness.

A variety of neurological disorders that lead to cognitive impairment have different types of advanced eye movement abnormalities. Within a certain range, the higher the cognitive workload of the paradigm, the higher the screening efficacy, e.g., antisaccades distinguish AD, aMCI, naMCI, and healthy controls, and pro-saccades do not. As a safe, reliable, and noninvasive examination method, advanced eye movement examination is superior to neuropsychological scales commonly used in clinical practice in terms of screening efficacy and differentiation, and can be applied to early screening for cognitive impaired disease. Current studies also show the great potential of this examination method in the early identification of these diseases, which is expected to become a biomarker for early clinical screening and clinical research of cognitive impaired diseases. However, this examination lacks standardized quantitative criteria and is not specific enough to distinguish between different subtypes of dementia, and its use in diseases in which cognitive impairment is a concomitant rather than the primary manifestation of the disease is questionable. More clinical studies should be conducted in the future to organically combine advanced eye movement examination with neuropsychological scale assessment and other testing methods to further early screen, identify, diagnose and monitor the evolution of Cognitive impaired disease and diseases concomitant cognitive impairment, which is of great clinical significance for early intervention and delaying disease progression.

## Author contributions

QL: Writing – original draft, Writing – review & editing, Conceptualization, Methodology. BD: Writing – original draft, Writing – review & editing. YJ: Conceptualization, Methodology, Project administration, Resources, Supervision, Validation, Writing – review & editing.
